# Relating microtensiometer-based trunk water potential with sap flow, canopy temperature, and trunk and fruit diameter variations for irrigated ‘Honeycrisp’ apple

**DOI:** 10.3389/fpls.2024.1393028

**Published:** 2024-05-24

**Authors:** Victor Blanco, Lee Kalcsits

**Affiliations:** ^1^ Department of Horticulture, Washington State University, Pullman, WA, United States; ^2^ Efficient Use of Water in Agriculture Program, Institute of Agrifood Research and Technology (IRTA), Lleida, Spain

**Keywords:** continuous measurements, fruit growth, plant-based sensors, precision irrigation, tree water status indicators, water potential

## Abstract

Instrumentation plays a key role in modern horticulture. Thus, the microtensiomenter, a new plant-based sensor that continuously monitors trunk water potential (Ψ_trunk_) can help in irrigation management decisions. To compare the response of the Ψ_trunk_ with other continuous tree water status indicators such as the sap flow rate, the difference between canopy and air temperatures, or the variations of the trunk and fruit diameter, all the sensors were installed in 2022 in a commercial orchard of ‘Honeycrisp’ apple trees with M.9 rootstocks in Washinton State (USA). From the daily evolution of the Ψ_trunk_, five indicators were considered: predawn, midday, minimum, daily mean, and daily range (the difference between the daily maximum and minimum values). The daily range of Ψ_trunk_ was the most linked to the maximum daily shrinkage (MDS; R^2^ = 0.42), the canopy-to-air temperature (Tc-Ta; R^2^ = 0.32), and the sap flow rate (SF; R^2^ = 0.30). On the other hand, the relative fruit growth rate (FRGR) was more related to the minimum Ψ_trunk_ (R^2^ = 0.33) and the daily mean Ψ_trunk_ (R^2^ = 0.32) than to the daily range of Ψ_trunk_. All indicators derived from Ψ_trunk_ identified changes in tree water status after each irrigation event and had low coefficients of variation and high sensitivity. These results encourage Ψ_trunk_ as a promising candidate for continuous monitoring of tree water status, however, more research is needed to better relate these measures with other widely studied plant-based indicators and identify good combinations of sensors and threshold values.

## 1 Introduction

Apples are ranked the fourth most cultivated fruit worldwide (FAOSTAT, 2022). The United States is the second most productive country, with an average annual production of 4.6 Mt. 69% of that production occurs in Washington State, where apple production entirely relies on irrigation (USDA, 2022). There, the adoption of dwarfing rootstocks, such as ‘M.9’, was important for increasing fruit yield and quality which consequently improved orchard profitability. This transition transformed traditional low-density planting orchards into modern high-density systems. Because of their smaller root biomass, dwarfing rootstocks are more vulnerable to water stress, soil water deficits and atmospheric demand (Lakso, 1994). Mild to severe uncontrolled water stress during orchard establishments in the first 2-3 years can reduce precocity and productivity, for mature trees, water stress can reduce fruit size (Robinson et al., 2013; Valverdi and Kalcsits, 2021).

Water limitations during early stages of fruit development can lead to an unbalanced fruit nutritional status which increases the occurrence of physiological disorders such as bitter pit in some cultivars like ‘Honeycrisp’ (Cheng and Sazo, 2018). On the other hand, over-irrigating can cause excessive vegetative growth, poor fruit quality such as lower firmness and soluble solids concentration, and make fruit more vulnerable to developing soggy breakdown and soft scald incidence (Robinson and Lopez, 2012). Moreover, overirrigation promotes excessively large apples for ‘Honeycrisp’ (diameter > 90 mm) which have been associated with increases in bitter pit incidence (Kalcsits et al., 2019; Lordan et al., 2019). That is why, irrigation strategies like regulated deficit irrigation that control tree vigor, and maintain optimum fruit nutritional status, size, and quality are used by growers in irrigated apple production regions (Reid and Kalcsits, 2020). However, implementation can be difficult since there is a lack of precise measures to improve irrigation management in these situations.

Precise irrigation management is needed to maximize fruit productivity and quality and save water resources. This requires careful monitoring of soil water availability and/or of tree water status. Although measuring soil water availability or estimating water-use based on environmental conditions remain the most commonly used approaches for irrigation scheduling, plant-based indicators of water status are increasingly being considered for irrigation decisions. There are many plant-based sensors that can continuously and directly measure real-time trees’ physiological responses and assess tree water status and fruit growth such as microtensiometers, sap flow sensors, thermoradiometers, and trunk and fruit dendrometers (Fernandez, 2017). All of them can be incorporated into decision-support systems for irrigation management and have shown a strong relationship with reference tree water status indicators such as the midday stem water potential measured with the Scholander pressure chamber and tree gas exchange (Fernandez, 2017; Noun et al., 2022). However, as has been previously reported by Garcia-Tejera et al. (2021), the benefits of continuously monitoring tree water status for irrigation management based on threshold values depend on the possibility of rapidly triggering irrigation when the threshold value is exceeded, which in commercial orchards is not always possible.

Microtensiometers consist of microelectromechanical pressure sensors that are embedded into the trunk (Pagay et al., 2014) and measure trunk water potential. Their measurements have been recently validated in different fruit trees and vines and under different environmental conditions and irrigation regimes with promising results (Blanco and Kalcsits, 2021; Lakso et al., 2022; Pagay, 2022; Blanco and Kalcsits, 2023; Conesa et al., 2023; Gonzalez Nieto et al., 2023a). Its continuous data acquisition may provide reliable and robust water status indicators that correspond well to commonly measured plant response traits such as the sap flow rates, the temperature of the canopy or the daily variations of the trunk and fruit diameter. These indicators may include midday, predawn, minimum, daily mean and daily range of trunk water potential. However, it must be said that these sensors are relatively expensive considering that their lifespan is not guaranteed after one season.

Sap flow sensors can estimate tree water use and consequently, irrigation doses, and have been described as a reliable tool for understanding plant hydraulic functioning (Alarcón et al., 2000; Burgess et al., 2001; Steppe et al., 2015). However, other authors have highlighted challenges associated with the installation process and wounding which can lead to different errors (Kumar et al., 2022) while others have recommended their use as a reference more than as an absolute value for calculating irrigation needs (Ballester et al., 2013).

Infrared radiometers installed above the canopy monitor canopy temperature. Increases in foliage temperature relative to air temperature can be an indicator of tree responses to water limitations (Jones, 2004; Mira-Garcia et al., 2022). Gómez-Candón et al. (2022) reported that this technology was a reliable indicator of water status in apple trees. However, the sensitivity of the indicators derived from the temperature of the canopy can be strongly affected by the age of the leaves or the development of vegetative flushes (Gonzalez-Dugo et al., 2014).

Trunk and fruit dendrometers continuously measure trunk and fruit diameters and have been extensively used by researchers to assess tree water status and fruit growth (Morandi et al., 2017; Ortuño et al., 2010). Trunk and fruit diameters increase during the night and either shrink or grow slowly during the day. Well-established indicators that use trunk diameter measurements include maximum daily shrinkage and growth rate which are sensitive to slight water deficits (De Swaef et al., 2009; Du et al., 2017; Blanco and Kalcsits, 2023). Fruit growth continuous measurements can be used to calculate growth rates which are parameters highly relevant as they are directly related to fruit yield and have shown good preliminary results for scheduling irrigation and determining water stress levels that do not penalize fruit growth (Fernandes et al., 2018). Fruit diameter is one of the most important characteristics for apples for accessing desired markets, along with fruit color. Although fruit diameter is largely determined by crop load (Serra et al., 2016; Gonzalez et al., 2020, Gonzalez et al., 2023), tree water status deeply affects fruit growth, as well as fruit quality and growers revenue (Ripoll et al., 2014). ‘Honeycrisp’ apples are within the top five most cultivated apples in the US and ‘Honeycrisp’ orchards show a much quicker investment payoff compared with other apple cultivars (Gonzalez Nieto et al., 2023b). They are the highest-priced apples in Washington State ($53.39 for a 40-pound box, 18.14 kg, Calvin et al., 2022), however, in order to reach those prices, high fruit standards regarding apple size and quality need to be met.

The aim of this work was to study the interaction, performance, and relationships of the water status indices derived from the continuous measures of the trunk water potential and compare them with other continuous, real-time, and easily automatable tree water status indicators that have been widely studied such as the sap flow rate, the canopy to air temperature, the trunk maximum daily shrinkage, the trunk growth rate, the fruit diameter variations and the relative fruit growth rate in a high-density commercial orchard of apple trees of the combination ‘Honeycrisp’/M.9.

## 2 Materials and methods

The experiment was conducted in a commercial apple orchard located in Grandview (Washington State, USA, 46° 18′ N, 119° 53′ W) from the end of June to the end of July 2022 spanning 31 days. The orchard (6.5 ha, elevation of 325 m, and North facing slope of 2°) was planted in 2009 with ‘Honeycrisp’/M.9 apple trees (*Malus × domestica* Borkh) in North-South oriented rows spaced 4.5 m and 0.8 m between trees (2778 trees ha^-1^) trained as a solaxe system. Trees had a crown area of 3.1 m^2^ at the beginning of the experiment (late June 2022). Full bloom and harvest dates were in late April and mid-September, respectively. The soil was characterized as a Hezel loamy fine sand (Sallato, 2023). Trees were drip irrigated with two drip lines per tree row with integrated emitters with a discharge rate of 3.78 L h^-1^ and a spacing of 45 cm.

**Figure 1:**
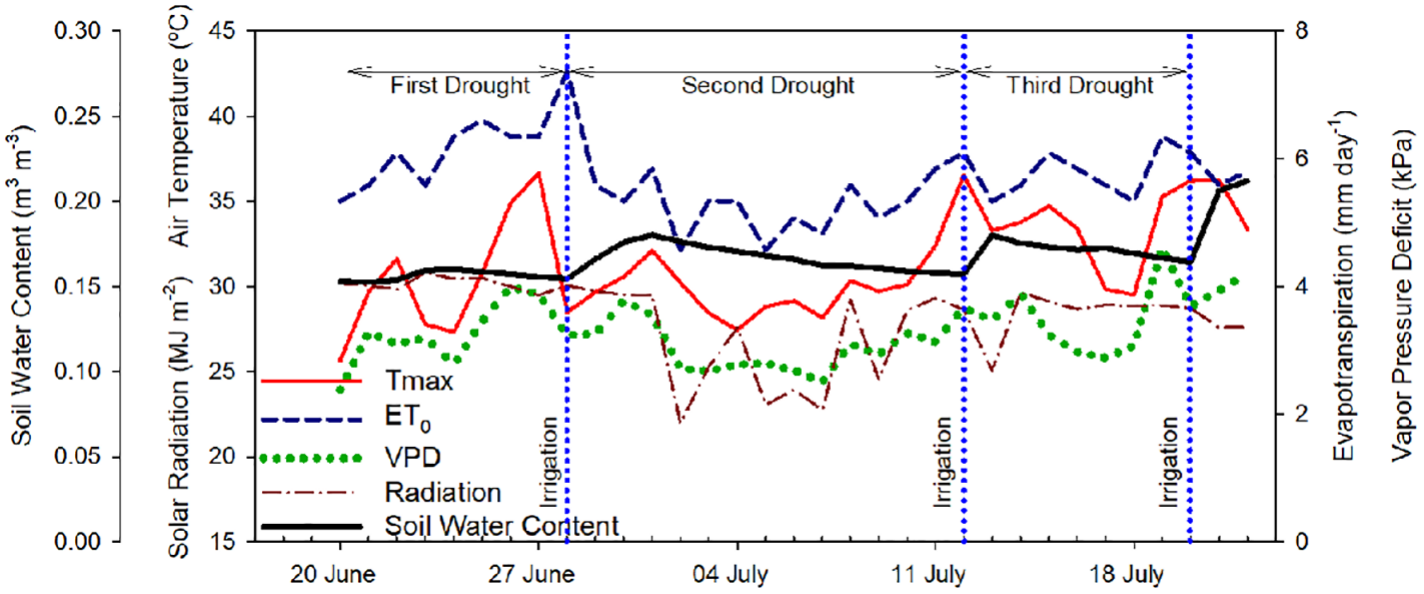
Evolution of the daily environmental conditions, maximum air temperature (Tmax), vapor pressure deficit (VPD), reference evapotranspiration (ET_0_), total solar radiation (MJ m^-2^), and soil water content (m^3^ m^-3^) from June 21^st^, 2022 to July 21^st^, 2022 in Grandview (Washington State, USA). Vertical blue dotted lines indicate the three irrigation events (June 28^th^, July 12^th^, and July 20^th^, 2022).

Environmental data (air temperature, relative humidity, total solar radiation, and reference evapotranspiration) were recorded by a weather station located close to the orchard and owned by AgWeatherNet (http://www.weather.wsu.edu; “Grandview station”). Daily air vapor pressure deficit (VPD) was calculated according to Allen et al. (1998). The mean VPD during the experiment was 3.3 ± 0.5 kPa and the maximum air temperature ranged between 25 and 37°C. Soil volumetric water content was measured at 0.3 m depth every 20 min with a capacitance domain sensor (5TM, Meter Group, Pullman, WA, USA). Soil water content values at field capacity and permanent wilting point were 0.25 and 0.10 m^3^·m^-3^, respectively (**Figure 1**).

The trees were irrigated three times during the 31-day experiment following commercial practices and according to the environmental conditions (crop evapotranspiration). The trees were going through three irrigation-drought cycles to evaluate the sensors’ response across a range of different environmental conditions and soil water availability (first drought cycle: June 20 – June 27 (8 days); second drought cycle: June 29 - July 11 (13 days); third drought cycle: July 13 – July 19 (7 days)). Initially, it was scheduled to apply each irrigation set every 7 – 8 days. However, during the second drought cycle, there were several cloudy days with values of VPD and maximum temperature below 2.5 kPa and 30°C, respectively, which decreased the accumulated reference evapotranspiration for the week, so the grower decided to postpone that irrigation set and expand the second cycle. Irrigation doses were automatically calculated by AgWeatherNet based on the crop evapotranspiration for high-density apple orchards. The first irrigation set was 4 h, while the second and third irrigation sets were 6 h. The irrigation timing varied during the study depending on the grower’s management. The first irrigation set was applied during the morning - early afternoon, the second irrigation set was applied during predawn - morning, and the third irrigation set was applied during the afternoon.

Three homogeneous and representative trees, in terms of trunk size, canopy volume and crop load, were selected to assess trunk water potential (Ψ_trunk_), the sap flow rate (SF), and the variations in trunk diameter. In two of those trees, the temperature of the canopy (T_c_) was also recorded. Fruit growth was measured in three apples for the same tree where all the sensors were installed. The total yield per tree, of the three trees monitored, was 55 apples of 72 mm of diameter (Sallato, 2023).

Ψ_trunk_ was recorded every 20 minutes using microtensiometers (FloraPulse, Davis, CA, USA). The microtensiometers were embedded into the trunk of the selected trees on the North side of the tree on a flat surface of the trunk at 0.5 m from the graft union. Five variables were obtained every day from the evolution of Ψ_trunk_ (midday, predawn, minimum, daily mean and daily range). Of those, three variables were extracted from the continuous measurements of trunk water potential: midday, predawn, and minimum trunk water potential. The daily mean was calculated as the average of all the values of Ψ_trunk_ recorded by the microtensiometers for one day. The daily range of the Ψ_trunk_ was calculated as the daily difference between the maximum and minimum values of Ψ_trunk_ recorded. Sap flow rates were continuously measured every 15 minutes with an exo-skin sap flow sensor (Model SGB19-WS, Dynamax, Inc., Houston, TX, USA) installed on the trunk of the tree in a region without branches. Hourly and daily values were then calculated from the continuous measurements. Canopy temperature (T_c_) was monitored every 15 minutes using infrared radiometer sensors (SPIP-IRT, Dynamax, Houston, TX, USA) with a precision of ± 0.5°C for air temperatures between 0 and 50°C. Midday T_c_ was compared to the air temperature (T_a_) in the orchard measured with an air temperature and relative humidity sensor (ATMOS-14, METER Group Inc., Pullman, WA, USA) installed within the tree row, 1 m above the tree canopy to calculate the difference between canopy and air temperature (T_c_-T_a_). Trunk diameter was monitored in the same three trees every 10 minutes using linear voltage differential pressure transducer dendrometers (LVDT, model DE-1T, Implexx Sense, Melbourne, Australia) with a 0.001 mm resolution. The sensors were installed in the trunk in a position in between the microtensiometer (located below the trunk dendrometer) and the sap flow sensor (located above) and with the same North orientation. Maximum daily shrinkage (MDS) was calculated as the difference between the maximum and the minimum trunk diameter recorded on each day. Trunk growth rate (TGR) was calculated as the difference between the maximum trunk diameter for the current and previous days (Goldhamer and Fereres, 2001). Fruit diameter was measured every 10 minutes by fruit dendrometers (LVDT, model FI-LT, Implexx Sense, Melbourne, Australia) and from those measurements, the daily fruit relative growth rate (FRGR) was calculated according to Scalisi et al. (2019), and the daily variations of fruit diameter (FDV) was calculated as the difference between the daily maximum and minimum fruit diameter. The three apples selected were at the same height (1.5 m from the soil), sun-exposed in the outer part of the canopy, and had a similar diameter at the beginning of the experiment of 35 – 37 mm.

Relationships between plant water status indicators were calculated from the data of all the days of the experiment and were explored through linear and non-linear regression analyses performed with SigmaPlot 12.5 (Systat Software Inc., San Jose, CA, USA). The sensitivity analysis of the tree water status indicators was calculated according to Goldhamer and Fereres (2001). The sensitivity (S) of each indicator was calculated by dividing Signal Intensity (SI) by the coefficient of variation (CV; the ratio of the standard deviation to the mean). SI was calculated according to Conesa et al. (2023) as the ratio between the values recorded on the three days before irrigation (drought cycle), and the values of the three days after the irrigation event.

## 3 Results and discussion

### 3.1 Trunk water potential

**Figure 2:**
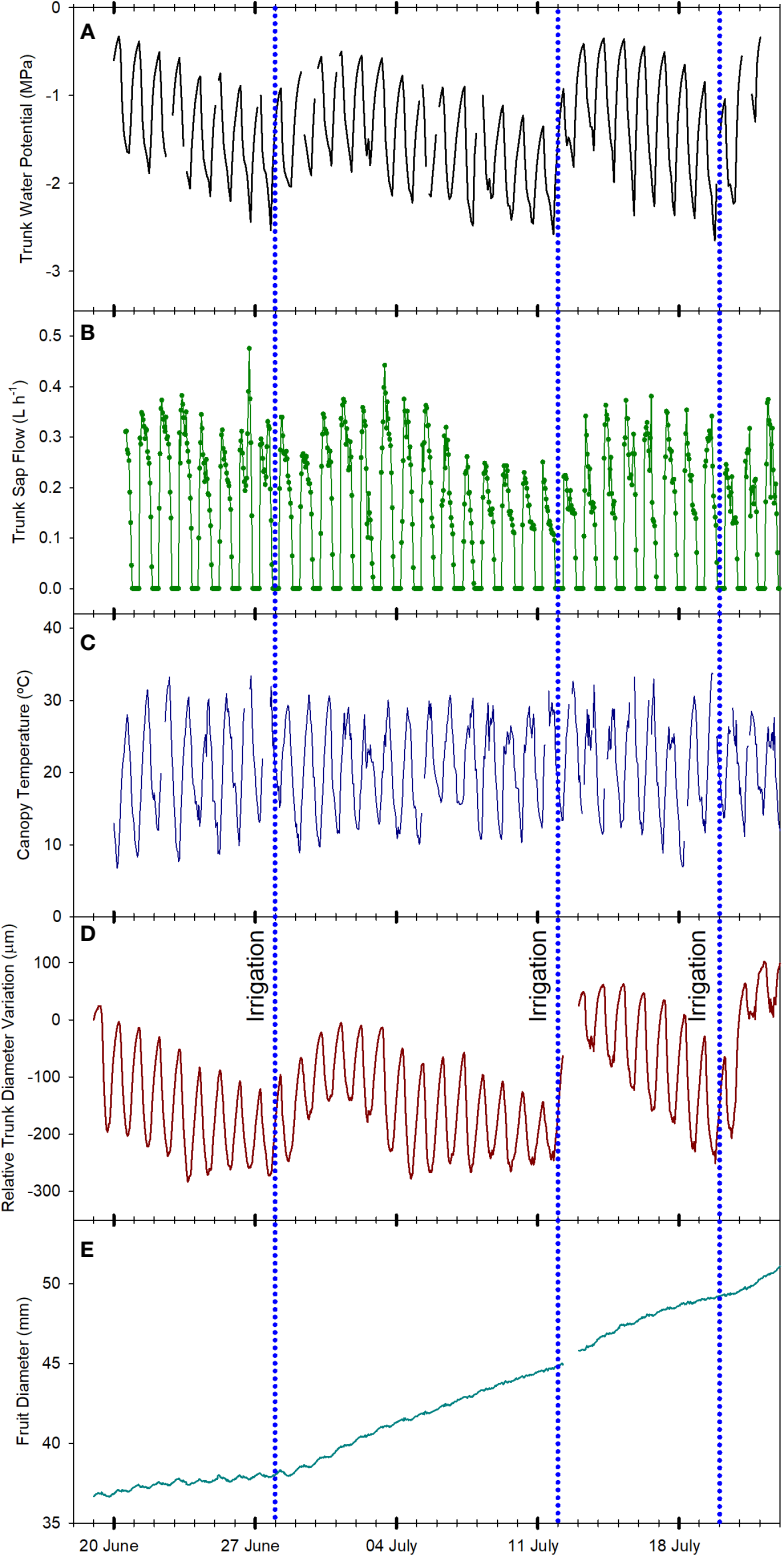
Evolution of the tree water status of one apple tree of the combination ‘Honeycrisp’/M.9 measured according to different physiological indicators: trunk water potential **(A)**, sap flow rate **(B)**, canopy temperature **(C)**, trunk diameter fluctuations **(D)** and fruit diameter growth **(E)** from June 21^st^, 2022 to July 21^st^, 2022 in Grandview (Washington State, USA). Vertical blue dotted lines indicate the three irrigation events (June 28^th^, July 12^th^, and July 20^th^, 2022).

**Figure 3:**
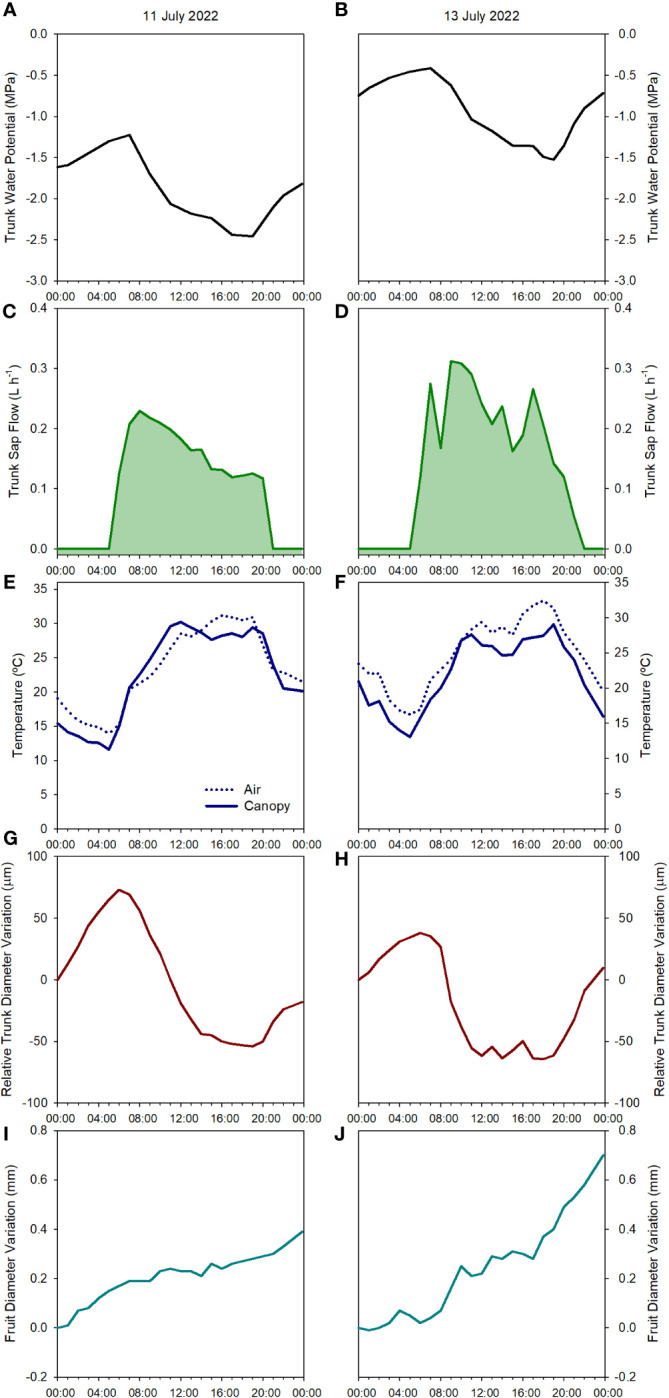
Diurnal evolution of the tree water status of one apple tree of the combination ‘Honeycrisp’/M.9 measured according to different physiological indicators: trunk water potential **(A, B)**, sap flow rate **(C, D)**, canopy and air temperature **(E, F)**, relative trunk diameter variations **(G, H)** and fruit diameter variations **(I, J)** on July 11^th^, 2022 (pre-irrigation **(A, C, E, G, I)** and July 13^th^, 2022 (post-irrigation **(B, D, F, H, J)**, in Grandview (Washington State, USA).

**Figure 4:**
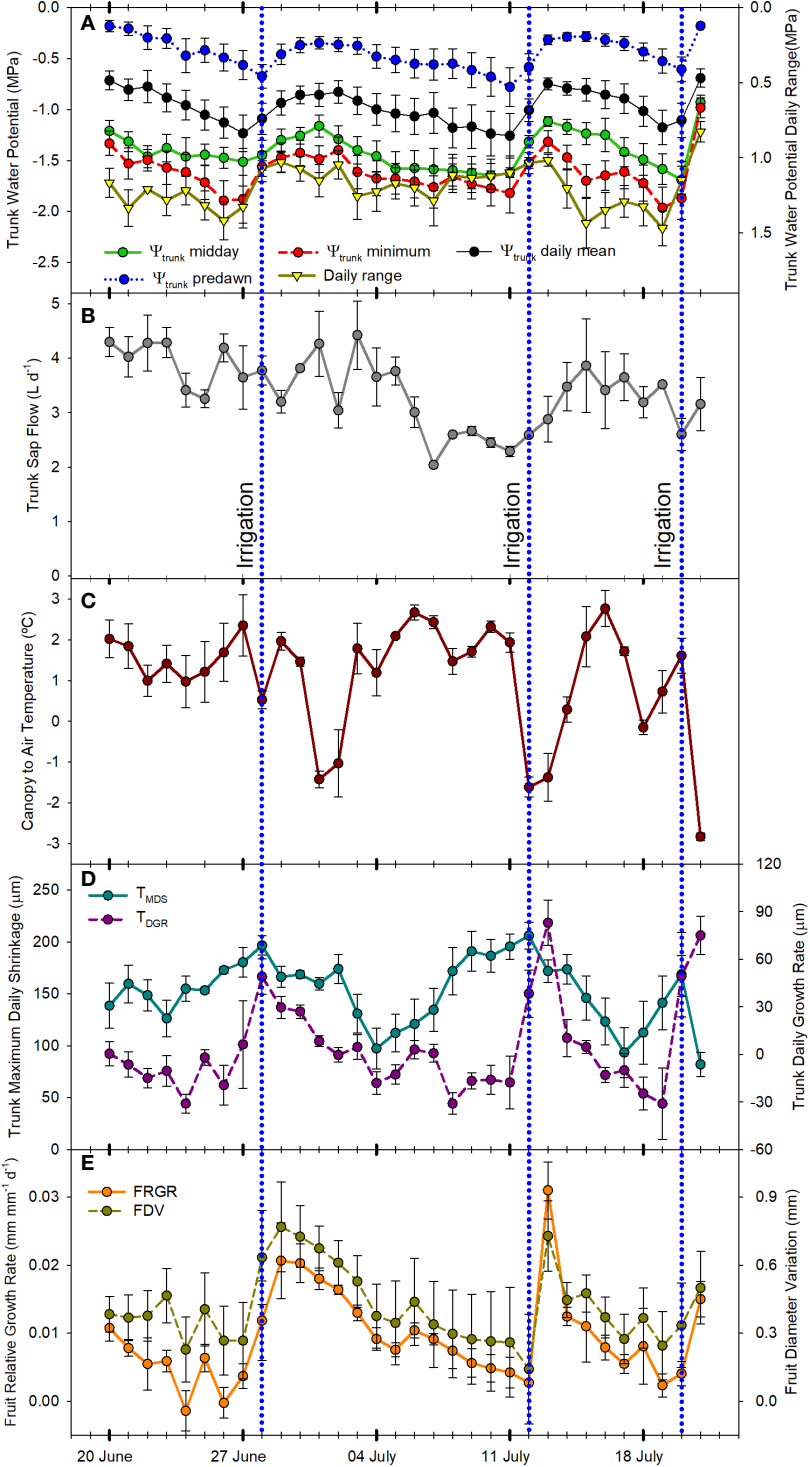
Mean value and standard error of midday, predawn, minimum, daily mean, and daily range trunk water potential **(A)** (n=3); total daily sap flow **(B)** (n=3); canopy to air temperature at midday **(C)** (n=2); trunk maximum daily shrinkage and daily trunk growth rate **(D)** (n=3); and fruit relative growth rate and fruit diameter variations **(E)** (n=3); from June 21^st^, 2022 to July 21^st^, 2022 in Grandview (Washington State, USA). Vertical blue dotted lines indicate the three irrigation events (June 28^th^, July 12^th^, and July 20^th^, 2022).

Trunk water potential (Ψ_trunk_) measured using microtensiometers has been recently reported as a reliable indicator of tree water status in apples (Lakso et al., 2022). This indicator was strongly related to the stem water potential measured with the pressure chamber, the reference indicator for assessing the water status of fruit trees (McCutchan and Shackel, 1992; Naor, 2000; Shackel, 2011). However, microtensiometers are not as responsive as the pressure chamber to detect fast changes in tree water status and can show a time lag (Lakso et al., 2022; Blanco and Kalcsits, 2023). In this experiment, all the indicators derived from Ψ_trunk_ followed a similar pattern (**Figure 2A**; **Figure 3A**; **Figure 4A**). Ψ_trunk_ rapidly changed after each irrigation event and midday and minimum daily Ψ_trunk_ increased by >0.4 MPa the next day, by 0.2 MPa for predawn Ψ_trunk_, and by 0.3 MPa for the daily mean. Recently, Gonzalez Nieto et al. (2023a) reported similar midday Ψ_trunk_ for ‘Gala’ apples under water restrictions in New York State. In contrast, the predawn values reported for this study in ‘Gala’ were not as equally affected by water stress as the midday or minimum values and they were never lower than -0.4 MPa. In the present study, predawn Ψ_trunk_ was clearly affected by the drought cycling with values lower than -1.0 MPa (**Figure 2A**) the days previous to the irrigation events. These differences might be attributed to the more demanding environmental conditions recorded in this experiment in the semi-arid climate of Washington State compared with more temperate conditions in New York State and to the cultivar used for this experiment. ‘Honeycrisp’ cultivar has shown lower water potentials than those reported for ‘Gala’ under water restrictions and fully irrigated (Valverdi et al., 2019).

The minimum values for Ψ_trunk_ on July 11^th^, 2022, before the irrigation was applied, were recorded during the afternoon (1700 – 1800 h, **Figure 3A**), a similar time of the day to those recorded in pears and vines under water stress in Washington State and Australia (Blanco and Kalcsits, 2021; Pagay, 2022) and slightly later than those recorded in nectarine trees in Spain (Conesa et al., 2023). Two days later on July 13^th^, 2022, the pattern was similar with maximum values at dawn and minimum values during the afternoon but with fewer negative values (**Figure 3B**). This rapid recovery of the trunk and stem water potential has been also observed in ‘Golden Delicious’/MM106 and ‘Gala’/G.11, with rapid changes of midday and minimum stem and trunk water potentials of more than 0.5 MPa (Doltra et al., 2007; Gonzalez Nieto et al., 2023a).

### 3.2 Sap flow

SF sensors recorded tree water use, with values that ranged between 2 and 4 L day^-1^ (**Figure 4B**). Similarly, Bhusal et al. (2019) reported sap flow rates for the medium maturing cultivar ‘Hongro’ on M.9 rates of 3 L day^-1^ for fully irrigated trees. However, they reported SF of 0.75 L day^-1^ for trees under no irrigation for 50 days and midday stem water potential below -2.5 MPa. In this experiment, for ‘Honeycrisp’ apples, the minimum value of SF measured was closer to 1.5 L day^-1^. For the days with midday trunk water potential below -1.7 MPa, it was observed a reduction in the rates of sap flow from values of 3.2 L day^-1^ to below 2.0 L day^-1^. These values were higher than those reported in ‘Fuji’ and ‘Golden Delicious’ under water restrictions (Liu et al., 2012; Bhusal et al., 2019). Moreover, it was also observed the strong effect that environmental conditions such as VPD, radiation, and air temperature had on tree transpiration rates (Liu et al., 2012). Thus, on July 2^nd^, 2022, although the minimum Ψ_trunk_ was -1.5 MPa, the low solar radiation (below 23 MJ m^-2^) and low evaporative demand (air temperature and VPD below 30°C and 3 kPa, respectively) caused the SF to decrease below 2.0 L day^-1^ (**Figures 1**, **2B**).

SF was the highest in the early morning with no differences observed at dawn on days before and after irrigation (0.2 L h^-1^). However, differences were first evident during the morning as the environmental conditions became more demanding. In the days before the irrigation, from dawn onwards, SF slowly decreased (**Figure 3C**). On the other hand, for the same tree in the days after the irrigation was applied the maximum daily values occurred between 0900 and 1100 h and then slowly decreased showing a sawtooth trend (**Figure 3D**). This pattern is similar to those reported in the cultivars ‘Braeburn’ (Green et al., 2003), ‘Fuji’ (Fernandez et al., 2008), ‘Nicoter’ (Ben Abdelkader et al., 2022), and ‘Mutsu’ (De Swaef et al., 2009). Rootstocks have also been reported to influence transpiration and SF (Cohen and Naor, 2002). Other apple cultivars grafted onto M.9, such as ‘Golden Delicious’ had similar SF values to those reported here, between 0.2 and 0.5 L h^-1^, while for the same cultivar on a more vigorous rootstock, SF was consistently higher (Li et al., 2002).

### 3.3 Canopy to air temperature

Canopy temperature (T_c_) is strongly affected by environmental conditions, so its use as an absolute value for assessing tree water status is not recommended (Idso et al., 1981; **Figure 2C**). However, when accounting for ambient conditions (T_a_), the use of thermal-based indices, such as the difference between canopy and air temperature (T_c_-T_a_) and the crop water stress index, can be reliable for assessing water stress in fruit trees (Gonzalez-Dugo et al., 2013; Ramirez-Cuesta et al., 2022; Blanco et al., 2023 The continuous monitoring of the T_c_-T_a_ has been described as the “heartbeat” of the tree water status (Mira-Garcia et al., 2022). In apple trees, Gómez-Candón et al. (2022) stated that T_c_-T_a_ is a sensitive indicator for many apple cultivars, and values between 1 and 2°C were related to midday stem water potentials between -1.6 and -1.8 MPa. Our results for ‘Honeycrisp’ followed a slightly different trend with T_c_-T_a_ at midday between 0.5 and 2.5°C (**Figure 4C**) when the environmental conditions were highly demanding and those days previous to the irrigation events (**Figure 3E**) but with negative values of T_c_-T_a_ at midday on the days after the trees were watered (**Figure 3F**) and on cloudy days with low evaporative demand, such as on July 2^nd^ when total solar radiation was below 25 MJ m ^-2^ (**Figure 1**). These results differ from those described for ‘Inored’ apples, in which positive values of T_c_-T_a_ were recorded in trees with soil water content values close to field capacity and midday stem water potential of -1.2 MPa (Gómez-Candón et al., 2022). Midday T_c_-T_a_ values higher than 2.5°C in ‘Honeycrisp’ apples occurred on days previous to the irrigation events, with minimum Ψ_trunk_ values similar to -2 MPa, and air temperature and VPD higher than 35°C and 4 kPa, respectively (July 19^th^ and 20^th^, 2022; **Figure 4C**). Similarly, the daily evolution of the T_c_-T_a_ was affected by environmental demand and soil water availability. On July 11^th^, 2022, (the day before the second irrigation event) the maximum positive difference between the canopy and air temperature was 2.9°C at midday and the most negative difference was -3.4°C during the night (**Figure 3E**). On July 13^th^, 2022, the day after the second irrigation event, all the values of T_c_-T_a_ recorded were negative, with the smallest difference at 1000 h, -0.4°C, and the largest at 1800h, - 5.6°C (**Figure 3F**). This pattern of negative T_c_-T_a_ values during the complete day agrees with those evolutions reported in ‘Fuji’ apples under full irrigation and mild water stress by Osroosh et al. (2015) with values similar to zero at midday and a second peak during the afternoon. The difference between T_c_ and T_a_ at 1800 h should be related to Tőkei and Dunkel (2005) in apple trees, where increases in transpiration rates during the afternoon occurred as a result of the decrease of water stress and opening of stomata.

### 3.4 Trunk diameter variations

As expected, daily trunk diameter fluctuations followed a similar pattern to Ψ_trunk_ and were responsive to changes in both soil water content and environmental conditions (**Figure 2D**). MDS increased when the trees were under water stress going from 120 µm to 250 µm during the drought cycles and decreased after each irrigation event (**Figures 3C** and **4D**). However, after reaching midday Ψ_trunk_ values of -1.8 MPa, MDS values did not continue to increase. Moreover, MDS values were also highly dependent on the environmental conditions so for consecutive days with similar values of minimum Ψ_trunk_, MDS varied by more than 25% (**Figure 4D**). Similarly, Du et al. (2017) reported for ‘Golden Delicious’ apple trees that MDS was strongly affected by environmental conditions. Thus, these both factors highlight the limitations of using absolute MDS values as a unique tree water status indicator. That is why, to decrease the variability of the MDS it has been recommended to express it relative to the MDS of a reference tree (non-stressed) which under commercial conditions might not be always suitable (Naor and Cohen, 2003).

For the TGR, negative values were recorded when the trees were under mild to severe water stress (**Figure 2D**) (midday Ψ_trunk_ values ranging from -1.1 to -1.7 MPa) and, as such, this indicator is not suitable for quantifying water stress in mature ‘Honeycrisp’ apple trees. However, TGR was sensitive to identifying the irrigation events applied, showing increases in trunk diameter of more than 40 µm for the days following irrigation (**Figure 4D**). Similar results were also reported in young ‘Cox Orange Pippin’ apple trees (De Swaef et al., 2009). Blanco and Kalcsits (2023), reported that variations in trunk diameter immediately followed changes in trunk water potential in pear trees. Increases in trunk diameter observed immediately after irrigation match the results of Bonany et al. (2000) in potted trees from the combination ‘Golden Delicious’/M.9. They suggested that young trees and trees on dwarfing rootstocks rapidly use and refill water stored in the trunk as a water source to maintain transpiration and fruit growth depending on the soil water content. In contrast, mature vigorous trees with a greater root volume such as ‘Golden Delicious’/MM106 do not follow this behavior (Doltra et al., 2007). As with the MDS, TGR values also showed a high variability on days with similar values of Ψ_trunk_ (**Figure 4D**), and it has been reported that TGR can vary significantly between cultivars, and rootstocks, and depend on factors such as tree vigor, crop load and age (Ortuño et al., 2010).

### 3.5 Fruit diameter variations

Daily FRGR was positive for most of the days of the experiment. Greater rates were recorded after irrigation, and when the evaporative demand was not excessively high (VPD < 3 kPa), while values close to 0 mm were recorded on days with ET_0_ values higher than 6 mm d^-1^. The daily FDV equaled the daily absolute growth since the maximum fruit diameter of the day generally matched the minimum fruit diameter of the next day (**Figures 3I, J**). Both, FRGR and FDV showed a similar trend to the TGR, emphasizing how both fruit growth and trunk water storage recovered when water supply increased (**Figure 4E**). The decrease in the daily FRGR and the FDV could be an early water status indicator to detect water deficit conditions for the trees (**Figure 2E**). Similar sensitivity of apple fruit diameter to water deficit has been reported in several cultivar and rootstock combinations as well as a range of environmental conditions (Morandi et al., 2017; Gonzalez Nieto et al., 2023a). Fruit growth occurred between evening and early morning for days before and after irrigation. No growth or a slight shrinking of the diameter was detected during midday or afternoon. These results agree with those reported by Boini et al. (2019) for ‘Imperial Gala’. When changes in fruit diameter were transformed into changes in mass (for ‘Honeycrisp’ apples, Diameter > 25 mm; Fruit Mass (g) = 0.07[Fruit Diameter (mm)]^2^ – 3.63[Fruit Diameter (mm)] + 59.41; Kalcsits et al., 2017), it was observed that the fruit growth was almost twice higher the day after the irrigation (0.7 mm, which equals ≈ 1.36 g fruit day^-1^; **Figure 3J**) than the day before (0.4 mm ≈ 0.69 g fruit day^-1^; **Figure 3I**).

### 3.6 Relations between trunk water potential and SF, Tc-Ta, MDS and TGR

**Figure 5:**
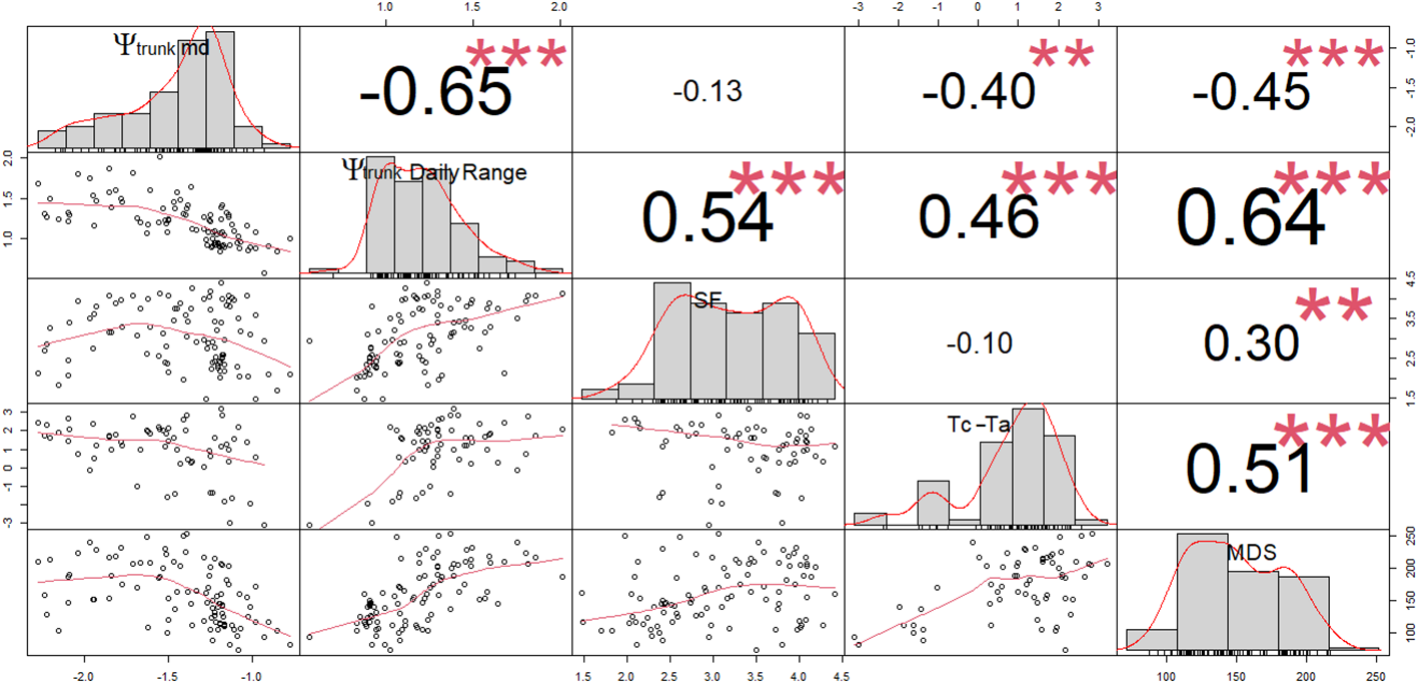
Correlation matrix (Pearson coefficients) for the linear regressions between midday trunk water potential (Ψ_trunk_ md; MPa), daily range of trunk water potential (Ψ_trunk_ Daily Range; MPa), sap flow rates (SF; L day^-1^), canopy to air temperature (Tc-Ta; °C) and maximum daily shrinkage of the trunk diameter (MDS; µm). ** and *** denote p-values < 0.01 and 0.001, respectively.

For ‘Honeycrisp’ apples, Ψ_trunk_ daily range showed the strongest and the most significant relationship with the SF, T_c_-T_a_, and MDS (based on a correlation analysis) of all the water stress indicators derived from Ψ_trunk_, followed by minimum and midday Ψ_trunk_ which had similar results (**Figure 5**).

Predawn Ψ_trunk_ did not show any significant relationship with SF, T_c_-T_a_, or MDS (p-values > 0.05). Several authors have also reported poor relationships between the predawn stem water potential and SF or MDS in apples, walnuts, and vines (Améglio et al., 1999; Intrigliolo and Castel, 2007; Liu et al., 2011). However, strong relationships have been reported in other tree fruit such as peaches and sweet cherries (Fereres et al., 1999; Livellara et al., 2011).

Based on linear regression analysis (**Figure 5**), the relationship between the indicators derived from the trunk water potential and MDS, SF and T_c_-T_a_ followed a polynomial pattern (**Table 1**). Thus, MDS reached its maximum values when midday and minimum Ψ_trunk_ were -1.8 and -2.0 MPa, respectively for ‘Honeycrisp’ apples. After that point, more negative tree water potentials were not related to greater MDS (**Figure 5**). This limitation has been previously reported in other fruit trees such as citrus, olive, stone fruits, and vines (Goldhamer et al., 1999; Ortuño et al., 2010; Blanco et al., 2018).

**Table 1:** Coefficient of determination (R^2^), and best fit quadratic equations [y = ax^2^ + bx +c] (quadratic coefficient (a), linear coefficient (b), and constant coefficient (c), number of data points (n) and p-value) between trunk water potential (midday, predawn, minimum, daily mean, daily range) measured by the microtensiometers and the maximum daily trunk shrinkage (MDS), difference between canopy and air temperature (T_c_-T_a_) and sap flow (SF) over the experiment.

	R^2^	a	b		c	n		p-value
Ψ_trunk_ *vs* MDS								
Midday	0.30	-110.55	-400.85		-172.96	93		<0.0001
Predawn	0.03	-70.80	-97.55		135.53	93		0.3157
Minimum	0.31	-80.42	-332.12		-151.98	93		<0.0001
Daily Range	0.42	-69.30	282.96		-75.85	93		<0.0001
Daily Mean	0.16	-103.08	-270.28		6.96	93		0.0004
Ψ_trunk_ *vs* T_c_-T_a_								
Midday	0.18	-1.71	-7.09		-5.64	62		0.0020
Predawn	0.05	0.15	-0.83		0.54	62		0.1824
Minimum	0.22	-1.73	-7.74		-7.00	62		0.0005
Daily Range	0.32	-4.70	15.40		-10.86	62		<0.0001
Daily Mean	0.10	-0.59	-2.52		-0.96	62		0.0423
Ψ_trunk_ *vs* SF								
Midday	0.14	-1.98	-6.42		-1.80	93		0.0016
Predawn	0.03	0.03	0.44		3.27	93		0.3580
Minimum	0.17	-1.34	-5.21		-1.58	93		0.0004
Daily Range	0.30	-0.85	3.65		-0.02	93		<0.0001
Daily Mean	0.08	-1.65	-3.95		1.01	93		0.0230

T_c_-T_a_ was more closely related to the daily range of Ψ_trunk_ than to the minimum and midday Ψ_trunk_ (**Table 1**). Ψ_trunk_ was the lowest during the afternoon when T_a_ was the highest. However, the greatest difference between T_c_ and T_a_ was often recorded at midday. In almond trees, Gonzalez-Dugo et al. (2012) also reported a strong relationship, which followed a second-degree polynomial function, between midday stem water potential and T_c_-T_a_ measured in the afternoon. A similar second-degree relationship between the stem water potential and thermal-based indicators has been reported in pear trees under similar environmental conditions (Blanco et al., 2023).

The relationship between midday Ψ_trunk_ and SF was parabolic (**Table 1**) with maximum values, above 4 L day^-1^, in the range between -1.2 and -1.7 MPa, and with values below 2 L day^-1^ when the trees were under severe water stress (< -2.0 MPa) or under nondemanding atmospheric conditions (> -0.9 MPa). De Swaef et al. (2009) reported a similar trend for ‘Mutsu’ apples, however the threshold value observed was -1.4 MPa, higher than that in this study. In grapevines, Patakas et al. (2005) reported that SF was 50% lower when the vines were under slight water stress.

Among the widely studied continuous indicators considered, MDS showed the strongest and most statistically significant relationship with Ψ_trunk_, T_c_-T_a_ ranked second, and SF was last (**Figure 5**). The strong relationship between Ψ_trunk_ and MDS might be due to the ability of both indicators to rapidly detect changes in the tree water status (Conejero et al., 2007; De Swaef et al., 2009). However, when the tree was under mid- to severe water deficit (< -1.8 MPa), Ψ_trunk_ was still able to detect and quantify water stress (until values below -2.5 MPa) while the MDS could not identify a situation of severe water stress. Ψ_trunk_ overcame the limitations of MDS and did not show any threshold limit to detect severe water stress in the range between -0.2 and -2.5 MPa. However, some limitations that have been reported to affect MDS such as the age of the tree, the crop load, or the phenological stage of the tree (Fernandez and Cuevas, 2010), might also affect Ψ_trunk_. Similarly, T_c_-T_a_ and SF have been reported to be strongly related to environmental conditions and were not recommended to detect slight water deficits or rapid physiological changes in response to water deficits (Ortuño et al., 2006; Mira-Garcia et al., 2022; Blanco et al., 2023).

**Figure 6:**
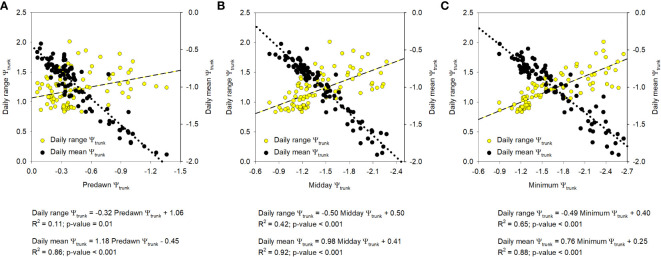
Relationship between the indices derived from the trunk water potential (Ψ_trunk_; MPa), daily range and daily mean, and the predawn **(A)**, midday **(B)**, and minimum **(C)** daily value. Each point corresponds to a specific tree and day (n=93).

The similar results obtained with the relationships between the SF, T_c_-T_a_, MDS, and TGR, and the midday and the minimum Ψ_trunk_ are explained since both (midday Ψ_trunk_ and minimum Ψ_trunk_) were strongly related (Ψ_trunk_ min = 1.18Ψ_trunk_ md + 0.06; R^2^ = 0.88). Predawn and midday Ψ_trunk_ were also strongly correlated (Ψ_trunk_ md = 1.07Ψ_trunk_ pd - 0.94; R^2 ^= 0.74). Daily maximum and minimum values of tree water potential, which correspond to predawn and midday -early afternoon respectively, have traditionally been used to determine tree water status in fruit trees (baselines) and threshold values to manage irrigation (Améglio et al., 1999; Naor et al, 1995; Shackel et al., 2021). However, the ability to continuously monitor Ψ_trunk_ with the microtensiometers provides an opportunity to explore other water potential-based indicators such as the daily mean and the daily range of Ψ_trunk_ that can integrate the tree water status for the whole day. These two indicators have not been widely explored when measuring the stem or leaf water potentials, but have the potential to be included in automated irrigation systems and are not as vulnerable as the midday Ψ_trunk_ to be affected by time lag problems. Both indicators, the daily mean and the daily range of Ψ_trunk_, were more related to the midday and minimum Ψ_trunk_ than to the predawn Ψ_trunk_ (**Figure 6**). The present work is the first report that assesses their adequacy as tree water status indicators in apple trees.

### 3.7 Relations between fruit growth and tree water status continuous indicators (Ψ_trunk_, SF, T_c_-T_a_, MDS and TGR)

Since fruit is the real target for growers, direct, continuous monitoring of fruit growth should be compared with the proposed tree water status indicators. The variability found among fruits for FRGR and FDV was similar to the variability reported in apple size within fruits from the same tree (Kalcsits et al., 2019).

The indices derived from the fruit growth were more related to the Ψ_trunk_ than to other tree water status indicators such as the MDS, SF, or T_c_-T_a_. According to our results, the daily mean of Ψ_trunk_ followed by the midday and minimum Ψ_trunk_ were the indicators derived from the trunk water potential that most closely corresponded to fruit growth rates (**Table 2**). Similarly, Boini et al. (2019) reported a strong relationship between midday stem water potential and changes in the FGR of ‘Gala’ apples. In this sense, recently, Gonzalez Nieto et al. (2023a) have developed the first logistic model that successfully relates Ψ_trunk_ with fruit growth for the cultivar ‘Gala’ in New York State.

**Table 2:** Coefficient of determination (R^2^), and best fit linear equations [y = bx +c] (linear coefficient (b), and constant coefficient (c), number of data points (n) and p-value) between relative fruit growth rate (RFGR) and daily fruit diameter variation (FDV) and trunk water potential (Ψ_trunk_) (midday, predawn, minimum, daily mean, daily range), sap flow (SF), difference between canopy and air temperature (T_c_-T_a_), maximum daily trunk shrinkage (MDS) and trunk growth rate (TGR) over the experiment.

	R^2^	b	c	n	p-value
FRGR *vs* Ψ_trunk_					
Midday	0.32	0.014	0.035	91	<0.0001
Predawn	0.21	0.012	0.019	91	<0.0001
Minimum	0.33	0.013	0.037	91	<0.0001
Daily Range	0.08	-0.008	0.022	91	0.0058
Daily Mean	0.32	0.014	0.030	91	<0.0001
**FRGR *vs* SF**	0.10	0.004	-0.003	91	0.0021
**FRGR *vs* T_c_-T_a_**	0.15	-0.002	0.012	91	0.0001
**FRGR *vs* MDS**	0.15	-6·10^-5^	0.021	91	0.0001
**FRGR *vs* TGR**	0.34	1·10^-4^	0.010	91	<0.0001
FDV *vs* Ψ_trunk_					
Midday	0.29	0.375	1.103	91	<0.0001
Predawn	0.22	0.345	0.683	91	<0.0001
Minimum	0.35	0.372	1.221	91	<0.0001
Daily Range	0.10	-0.254	0.792	91	0.0026
Daily Mean	0.29	0.386	0.964	91	<0.0001
**FDV *vs* SF**	0.17	0.132	-0.011	91	0.0001
**FDV *vs* T_c_-T_a_**	0.15	-0.058	0.492	91	0.0001
**FDV *vs* MDS**	0.12	-0.002	0.701	91	0.0009
**FDV *vs* TGR**	0.24	0.003	0.422	91	<0.0001

When trunk and fruit diameters were compared, there was a stronger relationship with the TGR than with the MDS, although there was a trend of lower FRGR when MDS increased. Bonany et al. (2000) described a linear relationship between MDS and FGR of young ‘Golden Delicious’ apples. However, that equation cannot be applied to our experiment because although the water potentials recorded in both experiments were similar, daily minimum values in the range between -0.7 and -2.2 MPa, MDS was larger, and RFGR were smaller. More work is needed to tune the relationship between the tree water status indicators and fruit growth during the full season and to assess for different cultivar/rootstock combinations how continuous monitoring of trunk water status can improve fruit yield and enhance fruit quality.

### 3.8 Sensitivity analysis

**Table 3** reports the sensitivity analysis of continuous tree water status indicators that were assessed. The index with the highest SI was T_c_-T_a_ (SI = 2.77) but the high CV decreased its sensitivity. Midday Ψ_trunk_ and the daily range of Ψ_trunk_ were the tree water status indicators with the highest sensitivity (S > 20) followed by the daily mean of Ψ_trunk_ (S = 17.53). Among the indicators derived from Ψ_trunk_, all showed similar SI, with the highest value observed for the daily mean Ψ_trunk_. However, the lowest CV was observed for midday Ψ_trunk_ which consequently increased its S. MDS had a high S, similar to the minimum Ψ_trunk_, while the highest CV and the lowest S were calculated for the TGR and the FRGR (S < 1). The CV values obtained for the MDS and the SF in ‘Honeycrisp’ apples were slightly lower than those reported by Wheeler et al. (2023) for the same indicators in ‘Aztec Fuji’ and ‘Escilate’, however, they followed a similar trend with the highest variability for SF, followed by the MDS and with the lowest variability for the stem water potential (0.09 – 0.11). Regarding the trunk water potential, similar results were reported by Conesa et al. (2023) who also highlighted the high sensitivity and low CV of Ψ_trunk_.

**Table 3:** Sensitivity analysis (Signal Intensity (SI), Coefficient of Variation (CV), and Sensitivity (S)) of trunk water potential (Ψ_trunk_) at midday, predawn, daily minimum, daily mean, and daily range, daily sap flow rate (SF), canopy to air temperature (T_c_-T_a_), maximum daily shrinkage (MDS), daily trunk growth rate (TGR), fruit relative growth rate (FRGR), and fruit diameter variations (FDV).

	Ψ_trunk_	Ψ_trunk_	Ψ_trunk_	Ψ_trunk_	Ψ_trunk_	SF	T_c_-T_a_	MDS	TGR	FRGR	FDV
	midday	predawn	minimum	range	mean						
**SI**	1.16	1.18	1.17	1.18	1.29	0.88	2.77	1.11	0.06	0.16	0.48
**CV**	0.05	0.09	0.08	0.06	0.07	0.11	0.35	0.07	1.10	0.21	0.10
**S**	22.72	12.68	13.80	21.26	17.53	7.75	8.00	14.83	0.05	0.79	5.03

**Figure 7:**
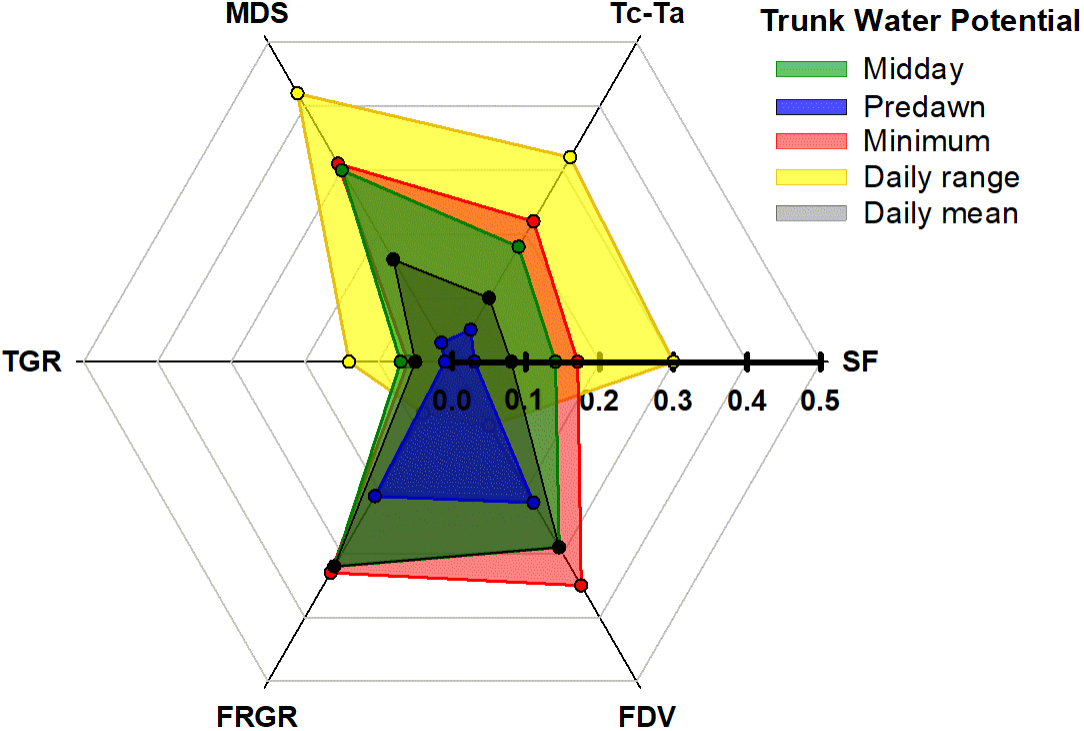
Coefficients of determination from the relationships between the trunk water potential indicators (midday, predawn, minimum, daily mean and daily range) and the sap flow rate (SF), the canopy to air temperature (Tc-Ta), the maximum daily shrinkage (MDS), the trunk growth rate (TGR). the fruit relative growth rate (FRGR). and the fruit diameter variations (FDV).

Therefore, the order proposed for the indicators according to their sensitivity was: midday Ψ_trunk_ = daily range of Ψ_trunk_ > daily mean Ψ_trunk_ > MDS = minimum Ψ_trunk_ > predawn Ψ_trunk_ > T_c_-T_a_ = SF > FDV > FRGR = TGR. Regarding the order of the indices derived from Ψ_trunk_ based on their relationship with the widely studied, traditional, continuous indicators, the general trend followed: daily range of Ψ_trunk_ > minimum Ψ_trunk_ = midday Ψ_trunk_ > daily mean Ψ_trunk_ > predawn Ψ_trunk_ (**Figure 7**). On the other hand, the daily mean Ψ_trunk_ and the minimum value of Ψ_trunk_ were the indicators more related to those indices derived from fruit growth.

## 4 Conclusions

Ψ_trunk_ is a reliable tree water status indicator for ‘Honeycrisp’ apple trees. Minimum Ψ_trunk_ and the daily range of Ψ_trunk_ were more sensitive and less variable than SF or T_c_-T_a_. Ψ_trunk_ was followed closely by MDS, which also showed low variability. On the other hand, TGR, the other indicator derived from the trunk diameter fluctuations, had the highest variability and consequently, was less sensitive. Concerning the changes in fruit size TGR, minimum Ψ_trunk_, and the daily mean Ψ_trunk_ were the indicators that better explained its variability, while the daily range Ψ_trunk_, SF, and T_c_-T_a_ were not as related. The minimum Ψ_trunk_ and the two new indicators derived from Ψ_trunk_ (daily range of Ψ_trunk_ and daily mean Ψ_trunk_) were able to identify the changes in tree water status and were not affected by time lags, which might affect midday Ψ_trunk_, so their used is preferred over it and the predawn Ψ_trunk_.

The close relationships found between the indicators derived from Ψ_trunk_ and MDS, SF, and T_c_-T_a_, make them promising plant-based indicators for precision irrigation management and encourage researchers to continue working with Ψ_trunk_ as a continuous indicator of tree water status. However, more work needs to be done to consider new indicators, threshold values, and cultivar-specific baselines to use Ψ_trunk_ alone, or in combination with other tree water status indicators in an independent irrigation system.

## Data availability statement

The raw data supporting the conclusions of this article will be made available by the authors, without undue reservation.

## Author contributions

VB: Conceptualization, Data curation, Formal analysis, Funding acquisition, Investigation, Methodology, Project administration, Resources, Software, Supervision, Validation, Visualization, Writing – original draft, Writing – review & editing. LK: Conceptualization, Data curation, Formal analysis, Funding acquisition, Investigation, Methodology, Project administration, Resources, Software, Supervision, Validation, Visualization, Writing – original draft, Writing – review & editing.
